# Evaluation of Bone Mineral Density in Children Conceived *via* Assisted Reproductive Technology

**DOI:** 10.3389/fendo.2022.827978

**Published:** 2022-02-07

**Authors:** Xinru Xia, Lingling Chen, Jing Wang, Xiang Yu, Li Gao, Yuan Zhang, Feiyang Diao, Yugui Cui, Jiayin Liu, Yan Meng

**Affiliations:** ^1^ State Key Laboratory of Reproductive Medicine, Center for Clinical Reproductive Medicine, First Affiliated Hospital, Nanjing Medical University, Nanjing, China; ^2^ Department of Pediatrics, First Affiliated Hospital, Nanjing Medical University, Nanjing, China

**Keywords:** bone mineral density, bone development, infertility, assisted reproductive technology, childbearing age

## Abstract

**Objectives:**

To investigate bone mineral density (BMD) differences between assisted reproductive technology (ART)-conceived children and naturally conceived (NC) children.

**Study Design:**

This retrospective cohort study included ART-conceived children and controls aged 1 to 12 years assessed with a follow-up protocol. Maternal and paternal background, birth condition, and growth and development indicators were analyzed.

**Results:**

The ART and NC groups exhibited differences in maternal and paternal childbearing age; maternal weight; maternal body mass index (BMI); maternal alcohol consumption; paternal smoking; delivery method; and serum zinc, iron, and lead levels. Multifactor analysis adjusted for relevant factors showed that paternal childbearing age and group significantly affected the BMD Z score. In the subgroup analysis, *in vitro* fertilization (IVF) (p=0.026) or intracytoplasmic sperm injection (ICSI) (p=0.008) had a positive impact on the BMD Z score. Male infertility only (p=0.010) or male infertility combined with polycystic ovary syndrome (PCOS) (p=0.026) may affect the BMD Z score. In the embryo transfer cycle subgroup analysis, compared with natural conception, both stimulation cycle fresh embryo transfer (p=0.019) and natural cycle frozen embryo transfer (p=0.006) had a positive effect on the BMD Z score.

**Conclusions:**

The BMD levels of the ART and control groups were generally in the normal range. Paternal childbearing age and the use of ART independently affected the BMD Z score of the offspring.

## Introduction

Infertility has become an increasingly common health problem, affecting approximately 48.5 million couples worldwide (1). Due to its high prevalence, it is regarded as a social disease by the World Health Organization. There are many reasons for infertility, and they vary from person to person. Assisted reproductive technology (ART) is one of the three main treatment strategies used for infertility. In recent decades, there has been great progress in ART, especially in the fields of fertility preservation, pre-implantation aneuploidy screening, uterine transplantation and mitochondrial replacement technology to prevent serious diseases, and previously incurable cases have been successfully treated (2–5). However, the risks of multiple birth, premature birth, very premature birth, low birth weight, very low birth weight, small for gestational age, congenital malformations, and birth defects are significantly increased in ART offspring (6–10). Bone density is the most sensitive early warning factor for osteoporosis. Osteoporosis is a serious disease, and there is inconsistent evidence regarding whether there is a difference in bone mineral density (BMD) in the offspring conceived by ART.

A study in 2015 showed that the speed of sound (SOS) level measured within 96 hours in ART preterm infants was lower than that measured in the naturally conceived (NC) group (11). However, another population-based study reported that there was no statistically significant difference in BMD between offspring aged 4 and 10 years who were born *via in vitro* fertilization (IVF) and fresh embryo transfer and those born *via* natural conception (12).

BMD is defined as the mass of bone mineral per unit volume. BMD is an indicator not only of the status of bone salt deposition but also of the status of bone development in children and adolescents (13). Childhood and adolescence are critical periods for bone development. Osteoporosis that occurs later in life is thought to have originated in childhood or adolescence (14). Thus, prevention and treatment of bone mineral deficiency in children is a key step to reducing the occurrence of osteoporosis in adulthood. The study of BMD in childhood has important healthcare significance.

BMD is affected by many factors, including genetic factors, birth state (15–17), age (18), sex (18), height (18, 19), nutrition (vitamin D and calcium intake) (19–21), pubertal status (19), life behavior (sun exposure, duration of breastfeeding or dietary pattern) (19, 22, 23), physical activity (19, 21), body composition (overall body mass, lean body mass or body fat mass) (24–26) and diseases (27–34).

Additionally, the health of offspring born after ART has been a focus of public attention. Whether ART itself or parental infertility affects the BMD of the offspring has not yet been reported.

Therefore, the purpose of this study was to determine whether the BMD of ART offspring is affected by infertility or ART itself and to explore the possible mechanism by which parental fertility and ART influence BMD to promote the bone health of ART-conceived children.

## Materials and Methods

### Subjects

The children who were recruited were conceived *via* ART in our center (ART group) or were NC (NC group) and came to our hospital for a physical examination in the Child Health Department from 2012 to 2015. The ART-conceived offspring included live-born infants from 2001 to 2014 in the Clinic Center of Reproductive Medicine of Jiangsu Province Hospital. The two groups were matched according to their age in months. Local institutional (First Affiliated Hospital of Nanjing Medical University) ethical approval (2012-SR-048) was obtained prior to data collection.

The inclusion criteria for the ART group were as follows: willingness to participate voluntarily and cooperatively, prepubertal status, singleton birth, full-term birth, *in vitro* fertilization and embryo transfer (IVF-ET) or ICSI as the ART method, infertility factors that included polycystic ovary syndrome (PCOS) and/or male factors (oligoasthenospermia, spermatogenic dysfunction or obstructive azoospermia), and embryo transfer cycle that was carried out *via* a fresh embryo being transferred in a stimulated cycle or a frozen embryo being transferred in a natural cycle.

The inclusion criteria for the NC group were as follows: willingness to participate voluntarily and cooperatively, prepubertal status, singleton birth, full-term birth, and natural conception.

The exclusion criteria for the ART group were as follows: illness or use of drugs, trauma or fracture, high-intensity sports training, family history of metabolic diseases, use of glucocorticoids by the mother during pregnancy, and donated sperm or eggs.

The exclusion criteria for the NC group were as follows: illness or use of drugs, trauma or fracture, high-intensity sports training, family history of metabolic diseases, and use of glucocorticoids by the mother during pregnancy.

In total, 84 individuals were included in the ART group, and 123 individuals were included in the NC group. Informed consent was obtained from a guardian of each participant included in this study.

### Follow-Up Process

Two weeks before follow-up, the subjects were notified by telephone of the time, place and contact person for the follow-up assessment.

The day before the follow-up assessment, the participants were called to confirm whether they were free to participate. The pregnancy information of the ART group was retrieved and printed from the Center of Clinical Reproductive Medicine system.

On the day of the follow-up, the specific follow-up procedure was explained, and an informed consent form was signed by the patient. The Pediatrics department conducted general physical examinations (height and weight), trace element analyses, and bone density tests. Parents filled out the questionnaire. After all the items were completed, the information was checked, and it was confirmed that there were no omissions; then, the data were entered into the computer.

### Ultrasound Scans

Using an Omnisense 7000P ultrasonic bone densitometer (Sunlight Medical Co., Ltd., Tel Aviv, Israel), after calibration by professional operators each day, the ultrasonic propagation velocity value (SOS) in the middle of the left tibia was measured by a standard method. The Z score of the SOS value of Asian children of the same age and sex was used as the standard.

### Measurement of Height and Weight

Height and weight were measured at the Department of Children’s Health Care by skilled personnel. The accuracy was within 0.1 cm and 0.1 kg, respectively. Body mass index (BMI)=weight (kg)/height (m)^2^.

### Determination of Trace Elements

Peripheral blood was taken from the child’s ring finger. The levels of zinc (Zn), copper (Cu), iron (Fe), calcium (Ca), magnesium (Mg) and lead (Pb) in peripheral blood were analyzed by an AA7000M atomic absorption spectrometer (East & West Analytical Instruments Co., Ltd., Beijing, China). The following normal ranges for different elements were applied: Zn (mg/L): 5–11.94; Cu (mg/L): 0.76–2.5; Fe (mg/L): 418.48–660.8; Ca (mg/L): 84–62.86; Mg (mg/L): 28.3–50.4; and Pb (μg/L): 0–100.

### Statistical Analysis

Data were analyzed using IBM SPSS 17.0 for Windows (SPSS, Inc., Chicago, IL, USA). In the single factor analysis, normally distributed continuous data are presented as the mean ± standard deviation (SD), and nonnormally distributed continuous data are presented as the median (interquartile range). Categorical data are presented as the frequency (%). Normally distributed continuous data were compared using an independent t test, and nonnormally distributed continuous data were compared using the Mann-Whitney U test. Unordered categorical data were compared using the chi-squared test, and ordered categorical data were compared using the rank-sum test. In the multifactor analysis, multiple linear regression analysis was used. Statistical significance was defined as p < 0.05.

## Results

### Single Factor Analysis of Bone Mineral Density-Related Factors

We evaluated whether there was a difference in indicators between the two groups (**Table 1**) and found that there was no significant difference in maternal height, smoking, paternal height, paternal weight, paternal BMI, alcohol consumption, full-term birth, amniotic fluid characteristics, birth length, birth weight, sex, age, height, weight, BMI, Cu, Ca, or Mg between the ART and NC groups. On the one hand, there was a significant reduction in Zn (p=0.009) and Fe (p=0.001) in the ART group compared with the NC group; on the other hand, maternal childbearing age (p=0.002), maternal weight (p=0.031), maternal BMI (p=0.021), paternal childbearing age (p=0.001), cesarean section rate (p=0.000) and Pb level (p=0.000) were significantly higher in the ART group than in the NC group. There was also a significant difference in maternal alcohol consumption (p=0.016) and paternal smoking (p=0.000) prevalence between the two groups.

**Table 1:** Single-factor analysis of bone mineral density-related factors.

	ART group (84)	NC group (123)	P value
Maternal childbearing age (years)	30 (27, 32)	28 (26, 30)	0.002**
Maternal height (cm)	160.5 (158.0, 165.0)	160.0 (158.0, 165.0)	0.694
Maternal weight (kg)	59.0 (53.0, 65.8)	55.0 (51.0, 61.7)	0.031*
Maternal BMI (kg/m^2^)	22.5 (20.4, 25.1)	21.3 (20.0, 23.4)	0.021*
Maternal smoking: Never	77 (91.7)	108 (87.8)	0.826
Ever	0	2 (1.6)	
Occasionally	3 (3.6)	2 (1.6)	
Still	0	1 (0.8)	
Missing	4 (4.8)	10 (8.1)	
Maternal alcohol use: Never	68 (81.0)	78 (63.4)	0.016*
Ever	3 (3.6)	0	
Occasionally	11 (13.1)	35 (28.5)	
Still	0	0	
Missing	2 (2.4)	10 (8.1)	
Paternal childbearing age (years)	32.5 (30, 35)	30 (28, 33)	0.001**
Paternal height (cm)	173.0 (170.0, 176.0)	173.0 (170.0, 176.0)	0.702
Paternal weight (kg)	70.0 (65.0, 76.0)	75.0 (66.5, 80.0)	0.181
Paternal BMI (kg/m^2^)	24.3 ± 2.99	24.8 ± 3.44	0.309
Paternal smoking: Never	20 (23.8)	31 (25.2)	0.000***
Ever	7 (8.3)	7 (5.7)	
Occasionally	10 (11.9)	20 (16.3)	
Still	41 (48.8)	52 (42.3)	
Missing	6 (7.1)	13 (10.6)	
Paternal alcohol use: Never	18 (21.4)	26 (21.1)	0.973
Ever	5 (6.0)	5 (4.1)	
Occasionally	47 (56.0)	60 (48.8)	
Still	9 (10.7)	18 (14.6)	
Missing	5 (6.0)	14 (11.4)	
Gestational age: Premature birth	8 (9.5)	6 (4.9)	0.421
Full-term birth	73 (86.9)	86 (69.9)	
Missing	3 (3.6)	31 (25.2)	0.000***
Delivery mode: Spontaneous delivery	17 (23.9)	54 (76.1)	
Caesarean section	63 (54.3)	53 (45.7)	
Missing	4 (20.0)	16 (80.0)	0.096
Amniotic fluid characteristic: Clean	74 (88.1)	92 (74.8)	
I°	0	5 (4.1)	
II°	1 (1.2)	3 (2.4)	
III°	1 (1.2)	1 (0.8)	
Missing	8 (9.5)	22 (17.9)	
Birth height (cm)	50.0 (50.0, 51.0)	50.0 (50.0, 51.0)	0.543
Birth weight (kg)	3.5 (3.0, 3.8)	3.5 (3.2, 3.8)	0.738
Sex: Male	46 (43)	61 (57)	0.482
Female	38 (38)	62 (62)	
Age (months)	35 (21.25, 46)	35 (20, 60.25)	0.381
Height (cm)	96.3 ± 13.85	98.6 ± 18.13	0.298
Weight (kg)	14.8 (12.4, 17.9)	14.9 (11.7, 19.0)	0.618
BMI (kg/m^2^)	15.8 (14.9, 17.0)	15.9 (15.0, 17.0)	0.662
Zn (mg/L)	5.54 (5.27, 5.74)	5.67 (5.38, 6.23)	0.009**
Cu (mg/L)	1.27 ± 0.167	1.26 ± 0.156	0.825
Fe (mg/L)	428.82 (424.83, 435.80)	434.16 (426.67, 445.92)	0.001**
Ca (mg/L)	66.72 (65.31, 68.16)	66.58 (64.87, 68.55)	0.625
Mg (mg/L)	36.38 ± 3.817	35.88 ± 3.478	0.361
Pb (μg/L)	47.00 (44.00, 49.00)	40.60 (32.21, 48.90)	0.000***
Z score	0.6 (-0.1, 1.1)	0.5 (-0.3, 1.0)	0.243

*p < 0.05; **p < 0.01; ***p < 0.001; ° degree.

The medians of the Z scores of the two groups were within the normal range, indicating that the total bone density level was not significantly abnormal in the ART or NC children.

### Multiple Linear Regression Analysis of Bone Mineral Density-Related Factors

After adjustments were made for the above confounding factors, paternal childbearing age (p=0.012) was still found to negatively affect the BMD Z score independently, which means that the higher the paternal age was, the lower the Z score would be. Grouping (p=0.004) positively affected the Z score of BMD, and the effect was greater than that of the father’s age. In other words, the Z score of the ART group was higher than that of the control group (**Table 2**).

**Table 2:** Multiple linear regression analysis of bone mineral density group category.

	β-value	95% CI		P value
Maternal childbearing age	0.027	-0.030	0.083	0.359
Maternal weight	-0.012	-0.057	0.034	0.611
Maternal BMI	-0.006	-0.136	0.125	0.932
Maternal alcohol consumption	-0.131	-0.320	0.057	0.301
Paternal childbearing age	-0.059	-0.104	-0.013	0.012*
Paternal smoking	-0.062	-0.188	0.064	0.333
Delivery mode: spontaneous delivery	-0.239	-0.588	0.111	0.179
Cesarean section(reference)				
Zn	-0.177	-0.477	0.124	0.247
Fe	0.001	-0.004	0.006	0.692
Pb	-0.010	-0.022	0.003	0.123
Group category: ART group	0.559	0.186	0.931	0.004**
NC group (reference)				

*p<0.05, **p<0.01.

### Subgroup Analysis of Bone Mineral Density-Related Factors

Next, we divided the ART group into different subgroups according to the ART method, infertility factors and embryo transfer cycles to find the source of the difference in BMD between the two groups.

In the ART method subgroup analysis (**Table 3**) IVF (p=0.026) and ICSI (p=0.008) had a positive impact on the Z score of BMD, and ICSI had a greater impact. This finding indicates that IVF or ICSI technology may affect the Z score of BMD.

**Table 3:** Multiple linear regression analysis of bone mineral density- ART methods.

	β-value	95% CI		P value
Maternal childbearing age	0.027	-0.030	0.084	0.350
Maternal weight	-0.014	-0.060	0.032	0.547
Maternal BMI	0.003	-0.132	0.137	0.966
Maternal alcohol consumption	-0.126	-0.316	0.065	0.194
Paternal childbearing age	-0.059	-0.105	-0.013	0.012*
Paternal smoking	-0.061	-0.188	0.065	0.339
Delivery mode: Spontaneous delivery	-0.235	-0.586	0.115	0.186
Cesarean section(reference)				
Zn	-0.167	-0.470	0.137	0.279
Fe	0.001	-0.004	0.006	0.715
Pb	-0.010	-0.023	0.003	0.121
ART methods: IVF (48)	0.494	0.059	0.929	0.026*
ICSI (36)	0.639	0.174	1.105	0.008**
NC (reference)				

*p < 0.05, **p < 0.01.

In the infertility factor subgroup analysis (**Table 4**), PCOS alone was not shown to affect the Z score of BMD compared with natural conception; however, male infertility (p=0.010) or male infertility combined with PCOS (p=0.026) positively affected the Z score, and the impact of male infertility combined with PCOS was greater. This finding indicates that paternal infertility may affect the BMD of offspring.

**Table 4:** Multiple linear regression analysis of bone mineral density—Infertility factors.

	β-value	95% CI		P value
Maternal childbearing age	0.025	-0.032	0.082	0.386
Maternal weight	-0.010	-0.056	0.037	0.677
Maternal BMI	-0.013	-0.149	0.122	0.846
Maternal alcohol consumption	-0.136	-0.327	0.055	0.160
Paternal childbearing age	-0.057	-0.103	-0.011	0.016*
Paternal smoking	-0.061	-0.188	0.067	0.348
Delivery mode: spontaneous delivery	-0.222	-0.575	0.130	0.214
Cesarean section (reference)				
Zn	-0.178	-0.480	0.124	0.246
Fe	0.001	-0.004	0.006	0.678
Pb	-0.010	-0.023	0.003	0.117
Infertility factors: PCOS (22)	0.443	-0.095	0.982	0.106
male infertility (52)	0.554	0.132	0.976	0.010*
male infertility combined with PCOS (10)	0.861	0.106	1.615	0.026*
NC (reference)				

*p < 0.05.

In the embryo transfer cycle subgroup analysis (**Table 5**), compared with natural conception, both stimulation cycle fresh embryo transfer (p=0.019) and natural cycle frozen embryo transfer (p=0.006) had a positive effect on the bone density Z score, and natural cycle frozen embryo transfer had a greater impact. This finding indicates that abnormal maternal hormone levels during fresh embryo transfer and frozen embryo technology may affect BMD.

**Table 5:** Multiple linear regression analysis of bone mineral density- embryo transfer cycles.

	β-value	95% CI		P value
Maternal childbearing age	0.023	-0.035	0.080	0.439
Maternal weight	-0.014	-0.060	0.032	0.545
Maternal BMI	0.002	-0.130	0.133	0.981
Maternal alcohol consumption	-0.129	-0.318	0.059	0.177
Paternal childbearing age	-0.057	-0.103	-0.011	0.015^*^
Paternal smoking	-0.063	-0.189	0.063	0.321
Delivery mode: Spontaneous delivery	-0.247	-0.596	0.103	0.165
Cesarean section (reference)				
Zn	-0.180	-0.480	0.121	0.239
Fe	0.001	-0.004	0.006	0.682
Pb	-0.010	-0.022	0.003	0.126
Embryo transfer cycles: fresh embryo transferred stimulation cycle (62)	0.481	0.082	0.881	0.019^*^
Frozen embryo transferred natural cycle (22)	0.765	0.228	1.303	0.006^**^
NC (reference)				

*p < 0.05, **p < 0.01.

## Discussion

ART has been used to treat infertility for more than 40 years. It has been confirmed that ART is associated with adverse perinatal outcomes, including premature birth, low birth weight, and increased risk of birth defects. A poor ART intrauterine environment and ART gamete manipulation, among other factors, are considered to be possible causes of poor ART pregnancy outcomes (35, 36). Therefore, the following question remains: as the child ages, is the growth and development of the ART-conceived offspring the same as that of the NC offspring?

Bone density characterizes bone development. Childhood is a critical period for bone development. Prevention and treatment of childhood bone mineral deficiency is important for reducing childhood rickets and osteoporosis in adulthood. Therefore, it is particularly important to assess the bone density of offspring conceived *via* ART.

Previous studies focused mostly on the BMD of ART-conceived offspring at birth or within a short period of time after birth. There is no long-term follow-up study on the bone development of ART-conceived offspring at preschool age. Therefore, this study focuses on the BMD of preschool-aged ART-conceived offspring. Whether there is a difference between the ART-conceived children and the NC offspring at the same age and the possible influencing factors were examined.

We found that the BMD Z scores of the offspring of the ART group were generally in the normal range and showed no obvious abnormality. This result is consistent with that of a 2007 study: There was no statistically significant difference between the BMD of the offspring conceived *via* IVF fresh embryo transfer at 4-10 years old and the NC offspring (12). Another study in 2015 showed that the SOS level measured within 96 hours in ART-conceived preterm infants was lower than that in infants in the NC group (11). The author suggested that this difference may be due to epigenetic changes in imprinted genes or other genes that undergo epigenetic modifications and participate in growth and the bone state. However, the number of participants in the study was relatively small, including only 37 ART-conceived infants (IVF or ICSI, no distinction between fresh embryos or frozen embryos) and 51 NC infants.

Our results revealed that the offspring of the ART group and the NC group had differences in maternal and paternal age, maternal weight, maternal BMI, maternal alcohol consumption, paternal smoking, delivery method, and serum zinc, iron, and lead levels.

The maternal and paternal ages in the ART group were higher than those in the NC group. This finding is consistent with previous studies (12, 37). Age is an independent risk factor affecting fertility; the older the age is, the higher the incidence of infertility. Thus, the childbearing age in the ART group was obviously higher than that in the NC group. Interestingly, after adjustments were made for the relevant variables in this study, maternal age no longer showed an independent effect on the BMD Z score. Instead, paternal age negatively affected the BMD Z score. A paternal childbearing age > 40 years is associated with an increased risk of spontaneous abortion (38–41), and advanced paternal age is associated with autosomal dominant disorders, such as Alport syndrome, achondroplasia and neurofibromatosis (42–46). There are also cohort studies and population studies showing that advanced paternal age is related to autism spectrum disorders and schizophrenia. A large prospective study of autism in Denmark with 1 million children found that the relative risk associated with a father’s age from 40 to 44 years was 1.6 (47), and a cohort study in Israel found that the odds ratio for a father’s age from 40 to 49 years was 5.75 (48). A large American study found that for every 10-year increase in paternal age, the relative risk of autism was 1.3 (49). Another study in Israel showed that the relative risk of having offspring with schizophrenia was 2.0 for fathers 45 to 49 years and 3.0 for fathers >50 years (50). Similar results have been observed in studies of other ethnic populations, including populations in Denmark, Sweden, and Japan (51–53).

Multifactor analysis with adjustments for relevant factors showed that paternal childbearing age and group category still significantly affected the Z score of the BMD. The possible reasons are as follows.

First, ART progeny may have subtle changes in the DNA methylation patterns of imprinted genes related to bone development. It has been reported in the literature that there is an increased risk of Beckwith-Wiedemwann syndrome in offspring conceived *via* ART (54–56). Although there is no description of the bone density of children with Beckwith-Wiedemwann syndrome, relatives of children with Beckwith-Wiedemann syndrome have been found to have higher childhood heights (12, 57). The phenotypic characteristics of being tall and having normal body weight confirm that the increase in the bone density Z score of ART-conceived offspring may be a subtle change in the DNA methylation pattern of imprinted genes related to skeletal development.

Second, ART-conceived offspring are exposed to supraphysiological doses of oestrogen *in utero*. The process of ART ovulation induces a high estrogen environment in the mother’s uterus, and estrogen promotes the development of the child’s bones. Estrogen can reduce the strain set point of the mechanical regulation system on the bone surface of the endosteum and increase the accumulation of bone in the cortex, causing thickening of the cortex (58, 59). In this study, to identify the possible reasons for the higher BMD of the ART offspring, the ART group was divided into different subgroups according to the ART method, infertility factors or embryo transfer cycle. In the ART group (62 cases), the BMD Z score was higher than that in the control group, verifying our hypothesis.

Skeletal development is affected by a variety of confounding factors, including genetics (parental bone development), socioeconomic background, nutritional status, and puberty. In our previous research, we collected data on the height of parents and found that the difference in height between the two groups was not statistically significant. Therefore, when we recruited participants, we chose families located in Nanjing, Jiangsu Province, to reduce the gap between family socioeconomic backgrounds and increase follow-up compliance. This study failed to accurately collect data on the eating habits of each participant. When we collected the study data, we collected information regarding the family’s dietary preferences in the questionnaire: vegetarian, meat-eaters, balanced diet or special eating habits. The vast majority of families consumed a balanced diet, and the offspring’s nutritional status was evaluated during the physical examination. It can be roughly considered that there was no significant difference in nutritional status between the two groups. Both groups of offspring were prepubertal children, thus eliminating the influence of pubertal sex hormones on bone development. We used inclusion and exclusion criteria to reduce selection bias, but there may still be potential uncontrolled confounding factors.

## Conclusion

Paternal childbearing age can independently affect the BMD Z score, and the higher the father’s age is, the lower the bone mineral density Z score will be. After adjustments were made for related confounding factors, the BMD Z scores of the ART group and the control group were still significantly different, that is, the bone density Z score in the ART group was significantly higher than that in the control group. The method of conception, ART indications and embryo transfer cycle all had a positive effect on the Z score of BMD.

### Limitations

The sample size of the subgroups still needs to be expanded to verify the results. However, additional longer-term, prospective follow-up multicenter studies are required to understand the increased risks among children conceived *via* ART.

## Data Availability Statement

The raw data supporting the conclusions of this article will be made available by the authors, without undue reservation.

## Ethics Statement

The studies involving human participants were reviewed and approved by Ethic Committee in First Affiliated Hospital of Nanjing Medical University. Written informed consent to participate in this study was provided by the participants' legal guardian/next of kin.

## Author Contributions

XX wrote the draft of the manuscript and performed the data analyses. LC, JW, and XY conducted the health tracking survey and acquired the data. LG and YZ recruited the participants. FD and YC reviewed and edited the manuscript. JL and YM designed the study. All authors contributed to the article and approved the submitted version.

## Funding

This work was funded by the National Key Research and Development Program of China (2016YFC1000204, 2017YFC1001300), the National Nature Science Foundation of China (81730041), and The National Key Research and Clinical Centre of Reproductive Medicine in Jiangsu Province of China (YXZXB2016001).

## Conflict of Interest

The authors declare that the research was conducted in the absence of any commercial or financial relationships that could be construed as a potential conflict of interest.

## Publisher’s Note

All claims expressed in this article are solely those of the authors and do not necessarily represent those of their affiliated organizations, or those of the publisher, the editors and the reviewers. Any product that may be evaluated in this article, or claim that may be made by its manufacturer, is not guaranteed or endorsed by the publisher.
